# European Society of Human Reproduction and Embryology annual meeting 2024

**DOI:** 10.1016/j.eclinm.2024.102781

**Published:** 2024-07-29

**Authors:** Ben Burwood

The European Society of Human Reproduction and Embryology (ESHRE) celebrated its 40th anniversary annual meeting in Amsterdam, The Netherlands, from July 7 to 10, 2024. With more than 1120 posters and oral presentations on offer, ESHRE 2024 celebrated successes from across the reproduction and embryology field. Here we report on some of the highlights from this year's conference.

## No increased risk of serious adverse events after fertility preservation in women with cancer

Data show that the average age of first-time mothers is increasing year-on-year. This increase in age has led to a growing number of women receiving a cancer diagnosis before they can fulfil their family goals, highlighting the critical importance of fertility preservation processes.

Older age and the impact of the cancer, in particular the proinflammatory and hypervascular environments and higher risk of thromboembolic events, might leave women with cancer at a greater risk of series adverse events following fertility preservation techniques. To assess this, Dr Julie Labrosse (Hopital Jean Verdier, Reproductive Medicine and Fertility Preservation, Bondy, France) presented the findings of a bicentric retrospective cohort study (Jan 1, 2014, to Dec 31, 2021) assessing the short-term safety of fertility preservation techniques in women with cancer.

1546 women (1678 treatment cycles) with cancer and 2798 women (2798 treatment cycles) who underwent fertility preservation for other indications were included in the study and assessed for the occurrence of seven serious adverse events: haemorrhage, ovarian hyperstimulation syndrome, thromboembolic event, infection, ovarian torsion, acute urine retention, and death. The women with cancer were significantly younger and had a significantly higher BMI compared with their comparators. Moreover, the women with cancer had a significantly fewer number of treatment cycles, most likely due to the need to start cancer treatment, but had a higher number of oocytes retrieved during the cycles compared with their counterparts.

In total, 17 intraperitoneal haemorrhages, eight reports of ovarian hyperstimulation syndrome, three infections, and one incidence of acute urine retention were recorded, and the authors concluded that women with cancer (0.36%) were at no greater risk of serious adverse event compared with their counterparts undertaking fertility preservation for other causes (0.82%; p = 0.061). When assessing the risk of adverse events by preservation technique, neither controlled oocyte stimulation (p = 0.077) nor in vitro maturation (p > 0.99) gave rise to an increased risk of serious adverse events in women with cancer. Of note, in an attempt to identify risk factors for serious adverse events across both indication groups, Dr Labrosse reported that women with a serious adverse event were more likely to be younger (29.8 years [SD 5.6] for those with a complication *vs* 32.9 years [5.4] for those with no complication; p = 0.0020) and more likely to have had a higher number of oocytes retrieved (13.6 oocytes [SD 9.1] *vs* 9.8 oocytes [7.4]; p = 0.0060) and frozen (10.8 oocytes [SD 6.7] *vs* 7.3 oocytes [6.0]; p = 0.0020).

The work by Dr Labrosse and colleagues is a positive step identifying the short-term safety of fertility preservation using oocyte stimulation and in vitro maturation in women with cancer. However, clinicians must be vigilant and heed the identified risk factors to ensure the best outcomes for their patients.

## An update on the Endometriosis Longitudinal Fertility Study (ELFS)

Around one in 10 women of reproductive age have endometriosis, with global estimates suggesting that 200 million people are affected. It is widely reported that endometriosis has a substantially impact on fertility, with 30–50% of women with endometriosis experiencing infertility. Moreover, women who are infertile are 6–8 times more likely to have endometriosis than those who are fertile, which illustrates the extent of the challenge caused by this disease. However, despite these stark data, information on disease management and fertility outcomes is limited, and mainly restricted to retrospective cohort studies. Laparoscopic surgery has been used to treat endometriosis, with some success in patients with mild disease; however, with poor evidence in women with moderate-to-severe disease and an 18% complication rate, questions remain over the use of this technique. Aiming to shed light on the natural history of women with endometriosis during their fertility journey, Dr Vanessa Ross (Royal Women's Hospital, Melbourne, VIC, Australia) presented the interim findings of the Endometriosis Longitudinal Fertility Study (ELFS), a multicentre, multinational longitudinal prospective cohort study.

Women younger than 38 years with untreated moderate or severe endometriosis and a current or future desire to become pregnant were included in the study. Following a baseline questionnaire, information on symptoms, conception attempts, surgical or other treatment initiated, and fertility investigations were collected from the participants using a specially designed smartphone application. The application sent personalised questionnaires to participants based on their cycle length and pregnancy status; made use of a branching logic algorithm; and had the ability to adjust to new circumstances, including alterations in cycle length, weight, and relationship status. Crucially, participants had to answer each question before moving on to the next element of the questionnaire, leading to impressive compliance and completion rates.

Dr Ross reported that information from 151 women (868 captured cycles) has been gathered, with 54 (36%; 193 conception attempts) women reported trying to conceive during the study (63 [33%] attempts via IVF or IUI *vs* 130 [67%] natural attempts). 33 women became pregnant (17% fecundity rate) resulting in 14 live births, eight ongoing pregnancies, and seven miscarriages.

The data presented were interim and recruitment is ongoing in Australia and Israel, but the results support the currently available literature. By providing granular data on disease trajectory and fertility outcomes stratified by method of conception and endometriosis management strategy, ELFS has phenomenal potential to elucidate on the best management strategies for women with moderate or severe endometriosis who desire to have children. We eagerly await the full findings.

## One to watch: the Copenhagen Pregnancy Loss study

More than 30 million pregnancy losses are reported to occur every year, effecting around 25% of pregnancies, yet, despite this, the mechanisms behind pregnancy loss are not fully understood. The previous idea that pregnancy loss is a result of fetal genetics does not paint a true picture. Although fetal genetics are understood to cause the loss in a majority of cases (50–60%), a non-genetic cause is thought to be responsible in a substantial number of losses (40–50%).

Dr Tanja Schlaikjær Hartwig (Amager and Hvidovre Hospital, Copenhagen, Denmark) reported on the going work of the Copenhagen Pregnancy Loss (COPL) study, which takes a trios approach to understanding pregnancy loss. The trios approach involves assessing the genetics, physiological, and sociodemographic characteristics of the mother and father in addition to analysing fetal genetics, using tissue from the pregnancy loss. To date, 2500 couples from four large hospital from across Denmark who had a missed miscarriage, ongoing spontaneous miscarriage, or anembryonic pregnancy have participated in the study, and, with plans to extend recruitment to 3000 couples, COPL has marked itself as a landmark investigation into the causes of pregnancy loss.

Seeking to improve our understanding of the causes and impact of pregnancy loss with a view to improving diagnosis, treatment, and prevention of pregnancy loss, the ongoing efforts of the team led by Prof Henriette Svarre Nielsen (Amager and Hvidovre Hospital, Copenhagen, Denmark) are to be applauded, and we look forward to seeing their discoveries.

